# Society Meetings

**Published:** 1917-05

**Authors:** 


### SOCIETY MEETINGS

Brief reports and announcements of meetings of societies of Western New York. and those of general scope, are requested from Secretaries. Copy should be on hand the fifteenth of the month. Full transactions will be published at cost of composition.

---

**The Medical Society of the State of New York** holds its 111th annual meeting at Utica April 24-26. The use of the present tense, as a compromise between the future correct at the time of writing and the past applicable to the appearance of the May issue, indicates the impracticability of reporting its transactions without delay till the June issue. We feel that every physician should hold membership in the State Society and take an active part in its work, and that we can show our loyalty in no better way than by leaving the report of its activities to the official organ.

---

The **American Proctologic Society** will hold its 19th annual meeting at the Hotel Astor, N. Y., June 4 and 5. An excellent program has been prepared and the profession is invited. The Secretary is Dr. C. F. Martin of Philadelphia.

---

The **Elmira Academy of Medicine** met April 11. Dr. A. H. Baker reported a case of Necrosis of the Clavicle, and Dr. W. A. DeWitt gave a paper on Local Anaesthesia.

---

**The Rochester Academy of Medicine**, Section IV, met April 11. Dr. Isaac Brewer spoke on Problems in Public Health Among the U. S. Troops on the Mexican Border.

---

**The Buffalo Academy of Medicine** has held the following meetings: Surgery, April 4, The Important Element in the Operative Treatment of Cases of Prostatic Obstruction, Dr. Arthur L. Chute, Boston. (To be publishel in a subsequent issue of this journal).

Medicine, April 11, Heart Block, Dr. Clayton W. Greene; Some Aspects of Syphilis in Children, Dr. Jacob S. Otto.

Obstetrics, April 18, Sterility—Causes and Treatment, Dr. Earl P. Lothrop.

---

**The American Gastro-Enterologic Assn.** will meet in Atlantic City, April 30 and May 1.

---

The annual meeting of **Alienists and Neurologists** will be held Monday, July 9 to Thursday, July 12, in the Red Room, LaSalle Hotel, Chicago, under the auspices of the Chicago Medical Society. Dr. George A. Zeller will act as chairman.

The program will be mailed June 28 with abstract of each paper. Contributors to the program are solicited. This is a society without a membership fee. Address, Secretary A. & N., room 1218, 30 N. Michigan Avenue, Chicago.

